# Psychedelics and fNIRS neuroimaging: exploring new opportunities

**DOI:** 10.1117/1.NPh.10.1.013506

**Published:** 2022-12-02

**Authors:** Felix Scholkmann, Franz X. Vollenweider

**Affiliations:** a University Hospital Zurich, University of Zurich, Biomedical Optics Research Laboratory, Department of Neonatology, Zurich, Switzerland; b University of Bern, Institute of Complementary and Integrative Medicine, Bern, Switzerland; c University Hospital of Psychiatry, University of Zurich, Neuropsychopharmacology and Brain Imaging, Department of Psychiatry, Psychotherapy and Psychosomatics, Zurich, Switzerland

**Keywords:** functional near-infrared spectroscopy, optical neuroimaging, psychedelics, altered states of consciousness

## Abstract

In this Outlook paper, we explain to the optical neuroimaging community as well as the psychedelic research community the great potential of using optical neuroimaging with functional near-infrared spectroscopy (fNIRS) to further explore the changes in brain activity induced by psychedelics. We explain why we believe now is the time to exploit the momentum of the current resurgence of research on the effects of psychedelics and the momentum of the increasing progress and popularity of the fNIRS technique to establish fNIRS in psychedelic research. With this article, we hope to contribute to this development.

## 1 Introduction

Although every human experiences two main states of consciousness on a daily basis (i.e., the waking state and the state of dreaming during sleep),^1^^,^^2^ there are many more tangible states of consciousness that can be located in a multidimensional state space consisting of different aspects of conscious experience.^3^ Altered states or nonordinary states of consciousness can be induced in various ways, such as training self-awareness while dreaming (lucid dreaming),^4^ using meditation techniques that can lead to deep meditative absorption,^5^^,^^6^ during life-threatening situations triggering a near-death experience,^7^^,^^8^ or by the intake of psychoactive substances (such as psychedelics).^9^^,^^10^ These nonordinary states of consciousness are of interest not only from a phenomenological^11^^,^^12^ and philosophical^13^^,^^14^ point of view but also with regard to the specific states of brain activity associated with them.^15^^–^^19^ Functional neuroimaging with its wide range of different techniques is an excellent way to investigate these specific states of brain activity.

The aim of this paper is to explain to the optical neuroimaging community as well as the psychedelic research community the great potential of using optical neuroimaging with functional near-infrared spectroscopy (fNIRS) to further explore the changes in brain activity induced by psychedelics.

## 2 Psychology and Neurobiology of Psychedelics

Classic psychedelics or hallucinogens compromise a class of psychoactive compounds that include (i) the naturally occurring indoleamines, such as psilocybin (4-phosphoryloxy-N,N-dimethyltryptamine) contained in a variety of fungi, and dimethyltryptamine (DMT) contained in the ayahuasca brew, (ii) the phenylalkylamines, such as mescaline derived from the peyote cactus, synthetic “amphetamines,” such as 2,5-dimethoxy-4-iodoamphetamine, and (iii) ergolines such as the semisynthetic lysergic acid diethylamide (LSD).^20^

Classic psychedelics induce an altered state of consciousness, characterized by profound changes in perception, mood, cognitive capacities, and self-experience, including transcendence of time and space.^11^ Given these intense mind-altering properties, plant-derived psychedelics have been used for millennia for spiritual and medicinal purposes.^21^^,^^22^

During the 1950s and 1960s, classic psychedelics (mainly LSD and psilocybin) were extensively investigated in psycholytic (i.e., repeated low doses) and psychedelic (i.e., one or two high doses) substance-assisted psychotherapy.^23^ Although these early studies used various psychotherapeutic techniques and had serious methodological flaws by contemporary standards, systematic reviews reported impressive improvement rates in various forms of depression, anxiety disorders, and alcohol-dependence.^24^^–^^26^ After psychedelics became schedule I substances in 1967, human research with psychedelics became severely restricted in most countries, leaving many questions unexplored.^27^

However, since the 1990s, several research groups have started to use modern neuroscience methods and concepts to characterize the psychological effects of psilocybin,^28^^–^^30^ DMT,^31^^,^^32^ and LSD.^33^^,^^34^ In addition, the study of the neuronal correlates of these psychological effects were resumed in healthy volunteers.^30^^,^^35^^–^^38^ These phase I studies provide evidence that classic psychedelics have rapid mood-enhancing properties, shift emotion processing in a positive direction, diminish self-boundaries, and reduce self-focus in combination with prosocial effects via modulation of neural circuits that are implicated in mood and affective disorders.^39^^–^^41^ Furthermore, psychedelics have been shown to produce lasting positive changes in psychosocial behavior in healthy subjects.^42^^–^^44^

Recent behavioral and neuroimaging studies demonstrate that psychedelics produce their psychological effects primarily via agonist action at serotonin $5\text{-}{\mathrm{HT}}_{2A}$ receptors in the brain,^15^^,^^45^^–^^47^ although the $5\text{-}{\mathrm{HT}}_{1A}$ receptor^48^ and modulatory downstream effects upon the GABAergic, dopaminergic,^49^^,^^50^ and glutamatergic^51^ systems are also implicated. Moreover, psychedelics have been shown to increase glutamate-driven neuroplastic adaptations in animals,^52^^–^^55^ which may provide a mechanism for the lasting beneficial outcomes reported in nonclinical and clinical populations.^39^

## 3 Resurgence of Psychedelic-Assisted Psychotherapy

In parallel to the research into the neuronal correlates of the psychedelic experience, the past decade has seen a resurgence and burgeoning research interest in the clinical potential of psychedelics in the treatment of various psychiatric disorders.^56^ Specifically, several recent pilot and a few controlled studies have demonstrated that psilocybin reduces substance use in alcohol- and nicotine-dependent patients^57^^–^^59^ and ameliorates both symptoms of anxiety and depression in major depression,^60^^–^^62^ treatment-resistant depression,^63^^,^^64^ and in advanced cancer patients^65^^–^^67^ for 3 to 6 months after administration of just one or two doses. Comparable results were reported for ayahuasca—a brew containing DMT—in major depression^68^^–^^70^ and for LSD in end-of-life psychological distress related to terminal illness,^71^ respectively.

These modern clinical trials provide new evidence for the safety, tolerability, and efficacy of the use of classic psychedelics in a supportive psychotherapeutic framework. It has been shown that psychedelic $5\text{-}{\mathrm{HT}}_{2A}$ agonists are rapidly acting and produce enduring beneficial effects after only one or two administrations.^56^^,^^72^ However, the underlying acute and delayed neurophysiological mechanism mediating these clinical effects is yet largely unknown.

Since psychedelics can have anti-inflammatory effects by modulating inflammatory pathways via novel mechanisms,^73^ they are currently also being explored for the treatment of neurodegenerative diseases,^74^^–^^76^ brain injuries,^77^ autoimmune diseases,^78^^,^^79^ as well as for chronic pain.^80^^–^^82^

## 4 Neuroimaging of Psychedelic Effects

Recent neuroimaging studies using electroencephalography (EEG), magnetoencephalography (MEG), and functional magnetic-resonance imaging (fMRI) in resting state and in combination with neuropsychological tasks in healthy subjects have advanced our understanding of the acute system-level effects and their association with behavioral changes.^83^ These discoveries provide a strategic scientific roadmap to further identify circumscribed neurobehavioral responses that may allow us to pinpoint the neuronal targets that may reflect specific symptom reductions in patients.

**Fig. 1:**
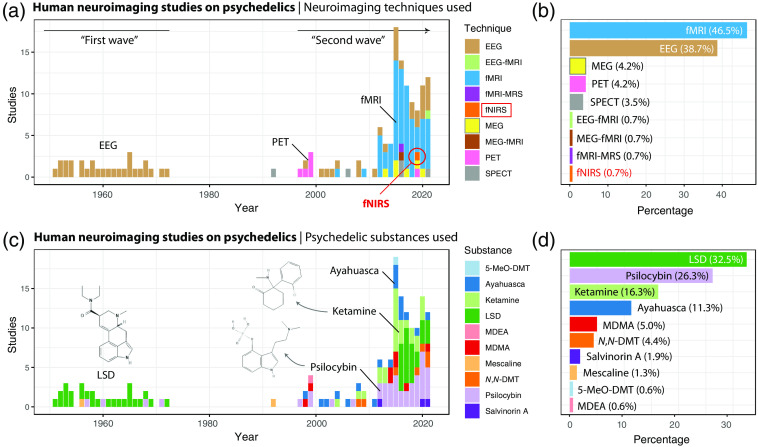
The development of human neuroimaging studies on psychedelics from the 1950s to 2020. Studies were identified via a search in PubMed and Google Scholar. A total of 141 studies were identified. Studies were only included when a modality of the acute effect of a psychedelic substance in a human was investigated. (a) and (c) visualize the number of studies as a function of the neuroimaging techniques or psychedelic substances used, respectively. Most of the studies employed fMRI (b) and investigated LSD (d). The “two waves” in the research development about human neuroimaging studies on psychedelics are clearly visible. In the first wave EEG was mainly used and the effect of LSD was investigated, whereas in the second wave, research opened up to other psychedelics and all available neuroimaging techniques were employed. Note: the listing contains also “salvinorin A,” which is a κ-opioid receptor agonist and considered a dissociative hallucinogen that can induce psychedelic-like effects. N,N-DMT: N,N-dimethyltryptamine, 5-MeO-DMT: 5-methoxy-dimethytryptamine, MDEA: 3,4-methylenedioxy-N-ethylamphetamine.

A recent review on human psychedelic research shows that during the 1950s and 1970s (i.e., the “first wave”),^17^ most neurophysiological studies into drug action were performed with EEG and primarily with LSD, whereas since the early 1990s and the recent renewed interest in the clinical application of psychedelics and related drugs (i.e., the “second wave”), researcher has begun to employ positron emission tomography (PET), photon emission computed tomography, and then later increasingly fMRI as well as EEG and MEG, to identify potential therapeutic targets primarily of psilocybin but also of LSD and DMT at the molecular and the neural system level (see Fig. 1). More recently, a few multimodal neuroimaging studies combining fMRI with MEG,^84^ magnetic resonance spectroscopy (MRS),^85^ and EEG^86^ have been also conducted. In addition, several neuroimaging studies have investigated the antidepressant of psychedelic-related drugs such as ketamine and 3,4-methylendioxymethampheamine (MDMA) in healthy subjects and clinical populations. In 2019, we explored in a single-subject pilot study the feasibility of investigating the effects of psilocybin using optical neuroimaging with fNIRS.^87^ The results of this pilot study showed that the application of fNIRS is safe and well tolerated during the induction of a psychedelic-induced altered state, and that this relatively new neuroimaging modality, particularly in combination with neuropsychological testing, may help unravel the therapeutic target of psychedelic drug action. This paper discusses new opportunities of fNIRS neuroimaging for psychedelic research.

## 5 fNIRS: Neuroimaging Technique with Much Progress and Increasing Popularity

**Fig. 2:**
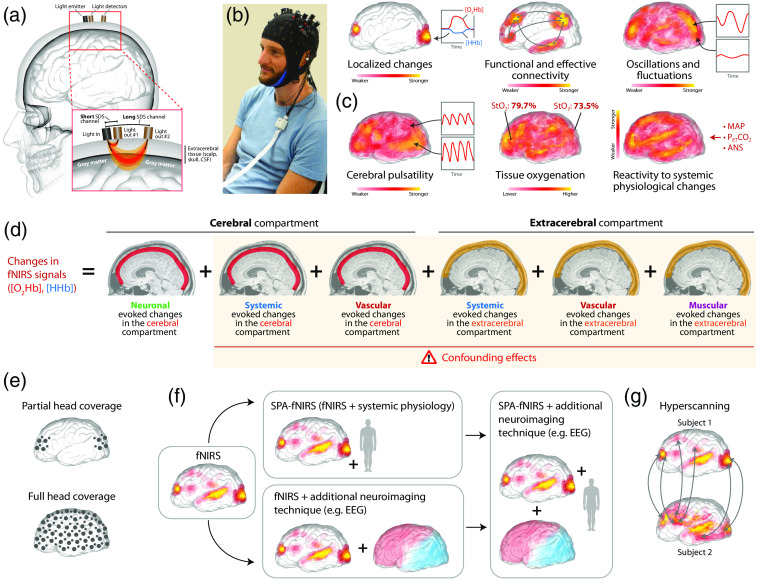
fNIRS neuroimaging: visualization of key aspects. (a) Illustration of a two-channel fNIRS measurement using a long and a short separation channel to enable a depth-resolved measurement specifically sensitive to the cerebral tissue layer. (b) A typical fNIRS headgear (covering the right and left motor cortices in this case). (c) The six main aspects that can be determined with optical neuroimaging employing fNIRS and NIRS-based oximetry. (d) The six main components of the fNIRS signal. (e) Two typical fNIRS instrumentations with regard to the spatial positioning of the light sources and detectors on the head. (f) Visualization of current trajectories of fNIRS development. (g) The fNIRS hyperscanning approach.

Over the last decades, optical neuroimaging with fNIRS is rapidly gaining popularity in neuroscience, which can be seen in the exponential number of articles published^88^^,^^89^ and an increased number of commercially available fNIRS devices. Both fNIRS and fMRI are techniques that measure brain activity indirectly by determining the changes in vascular hemodynamics and oxygenation induced by neuronal activity (neurovascular coupling). fNIRS is based on the principle that near-infrared light (with at least two different wavelengths) is shown in the head by placing light emitters on the scalp and detecting the diffusely back-scattered light at specific distances apart [Fig. 2(a)]. This allows to perform the spectroscopic determination of changes in the concentration of oxyhemoglobin ($\left[O_{2}\mathrm{Hb}\right]$), deoxyhemoglobin ([HHb]), and total hemoglobin $\left([\mathrm{tHb}]=[O_{2}\mathrm{Hb}]+[\mathrm{HHb}]\right)$.^90^ The measurement determines the color of the blood (light red versus dark red: oxygen-rich versus oxygen-poor blood) as well as the color intensity (high color intensity: higher hemoglobin concentration). The light detectors and emitters are normally mounted on a cap [Fig. 2(b)] and measurements can be made independent of body position and even in moving subjects.^91^

The fNIRS signals are rich in information and there are several ways to perform the measurements and analyze the data. In general, fNIRS can be used to measure six physiological aspects [Fig. 2(c)]:

(i) localized stimulus- or task-induced changes in cerebrovascular hemodynamics and oxygenation;^92^^–^^94^
(ii) functional and effective connectivity of localized evoked or resting-state changes in cerebral hemodynamics and oxygenation;^95^^–^^97^
(iii) oscillations and fluctuations of tissue hemodynamics and oxygenation [e.g., changes in Mayer wave power (around 0.1 Hz)^98^];
(iv) cerebral pulsatility (i.e., cardiac activity-induced changes in the fNIRS signal^99^^–^^102^);
(v) cerebral tissue oxygenation [which can be measured as relative oxygenation changes with respect to a baseline (the option available in most of the commercial fNIRS devices on the market), or absolute tissue oxygenation (i.e., near-infrared spectroscopy-based oximetry based on frequency-domain, time-domain, or specific types of continuous-wave domain near-infrared spectroscopy techniques)];^103^^,^^104^ and
(vi) reactivity of extracerebral and cerebral tissue hemodynamics and oxygenation to systemic physiological changes (i.e., measuring aspects of cerebrovascular reactivity, cerebral autoregulation, and autonomic cerebrovascular control).^105^^,^^106^

When aiming to measure brain-activity-related changes in cerebrovascular hemodynamics and oxygenation with fNIRS, one needs to be aware that the measured fNIRS signal generally comprises six components [Fig. 2(d)]:^90^^,^^107^^,^^108^ the first three have their origin in the cerebral tissue compartment, and the other three in the extracerebral tissue compartment. To detect brain activity–related changes in vascular hemodynamics and oxygenation due to neurovascular coupling, only changes happening in the cerebral compartment are of interest (i.e., the first component). Systemic physiology affects both tissue compartments and can lead to changes in hemodynamics and oxygenation, for example induced by changes in the cardiorespiratory state or autonomic nervous system activity. Furthermore, spontaneous fluctuations in tone of blood vessel walls (vasomotion) cause another component also present in both tissue compartments.^109^ Finally, muscular evoked changes can be induced by the activity of the temporal muscle on the head.^110^^,^^111^ The non-neuronal driven components (i.e., components 2 to 6) are a challenge for fNIRS since they may mimic typical fNIRS signal changes normally observed due to an increase (or decrease) of brain activity (a “false positive”), or they may mask a neuronal-induced hemodynamic response so that it is not detected anymore (a “false negative”).^107^^,^^112^ Although the significance of non-neuronal drivers of the fNIRS signal changes is increasingly recognized, these non-neuronal drivers (e.g., systemic and vascular ones) are also increasingly in the focus in the field of fMRI due to their impact on the BOLD signal.^113^^,^^114^

Optical neuroimaging with fNIRS can be performed either by measuring regions of interest with a partial coverage of light emitters and detectors or using a full head coverage [Fig. 2(e)]. Measurements with different source–detector distances (short and long ones) enable depth-dependent measurements and reduction of the influence from extracerebral tissue layers,^115^^–^^117^ and when combined with a high-density coverage of light emitters and detectors also to perform a tomographic reconstruction of cerebrovascular hemodynamics and oxygenation (also termed “diffuse optical tomography” or “near-infrared optical tomography”).^118^^,^^119^

As far as current trends in fNIRS neuroimaging are concerned, there is a development toward using fNIRS in combination with the measurement of systemic signals [an approach termed “systemic physiology augmented functional near-infrared spectroscopy” (SPA-fNIRS);^92^^,^^120^ for a review see Ref. 108], or combining fNIRS with other neuroimaging techniques, such as EEG,^121^^–^^123^ fMRI,^121^^,^^124^ or PET.^125^^–^^128^ Ideally, both approaches can then be combined [Fig. 2(f)]. In addition, future commercial fNIRS devices will probably also work with even more wavelengths (“broadband NIRS”, bNIRS), which will enable the direct measurement of metabolic parameters (e.g., the concentration of cytochrome-c-oxidase).^129^ Moreover, time-domain fNIRS devices are expected to play an increasingly important role,^130^^–^^132^ the promising interferometric NIRS technology is currently being further developed and explored for fNIRS applications,^133^^–^^135^ and the combination of fNIRS with diffuse correlation spectroscopy offers great potential for detailed measurement of hemodynamic changes.^136^^,^^137^ Another trend is the performance of fNIRS measurements on two or more people at the same time (the “hyperscanning” approach) [Fig. 2(g)].^138^^–^^140^

## 6 fNIRS Neuroimaging as a Promising New Technique for Psychedelic Neuroscience

Optical neuroimaging with fNIRS has specific features that make it a quite unique approach to measure neurovascular and neurometabolic changes associated with brain activity. Compared to the other neuroimaging techniques, fNIRS has its advantages but also limitations.

The main advantages are that fNIRS

(i) enables the measurement of a broad set of parameters related to cerebral hemodynamic, oxygenation, and metabolism [especially when specific advances technical NIRS implementation are used; see Fig. 2(c)];
(ii) is more cost-effective compared to the purchase and operation of an fMRI scanner;
(iii) does not produce disturbing noise like an fMRI (and thus avoiding stress induced by the noise in the subjects);
(iv) is much more robust against movement artifacts than an fMRI measurement—it can be used even when the subject is moving (an aspect that makes it ideally suited for psychedelic research since under the influence of a psychedelic substance the subject can feel and urge to move the body);
(v) allows measurement of the subject in different body positions (fMRI normally allows only the supine position);
(vi) makes it possible to perform relatively long measurements (several hours) which could cover the whole dynamics of the psychedelic experience;
(vii) is ideally suited for multimodal measurements combining different types of neuroimaging as well as to combine fNIRS neuroimaging with monitoring systemic physiological activity (the SPA-fNIRS approach); and
(viii) enables neuroimaging to be performed in many subjects in parallel (hyperscanning), ideally suited to investigate the impact of the group-setting and personal interactions during psychedelic sessions.

With regard to limitations, the main limitations of fNIRS neuroimaging are that

(i) the light penetration is limited so that only tissue hemodynamic, oxygenation, and metabolism originating from the cerebral cortex can be measured;
(ii) the measured fNIRS signals comprise different components [Fig. 2(d)] that need to be separated in order to enable a correct physiological interpretation of the signals;
(iii) wearing the fNIRS cap can be uncomfortable (but this can be improved considerably by optimizing the cap accordingly), which is particularly relevant for longer measurements or experiments where the subject should not be stressed by additional factors (e.g., during a psychedelic experience);
(iv) the fNIRS signal processing and data analysis are complicated, and the related standardization is currently still subject of discussion and development.^141^

**Fig. 3:**
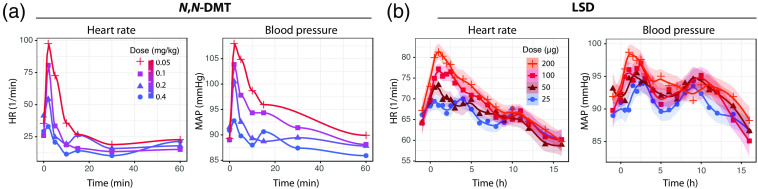
Examples of changes in cardiovascular and autonomic nervous system activity in humans induced be the intake of psychedelics. (a), (b) Changes in heart rate and blood pressure receiving (a) doses of 0.05, 0.1, 0.2, and 0.4  mg/kg N,N-DMT^32^ and (b) doses of 50, 100, and 200  μg LSD.^142^ New visualization of the data presented in Figs. 9 and 10 of the paper of Strassman and Qualls, and Fig. 3 of the paper of Holze et al., respectively. Shown are mean values (a), (b) as well as regression functions and the confidence interval for the regression functions [(b); own calculation].

Psychedelics induce changes in the activity of the autonomic nervous system, cardiorespiratory, and cardiovascular system^32^^,^^142^^,^^143^ (Fig. 3) in a subject- and substance-dependent manner. These systemic physiological changes will influence the fNIRS measurements and it is recommended to use a depth-resolved measurement technique, the SPA-fNIRS approach, and a careful as well as detailed analysis of the interplay between cerebral fNIRS data and systemic physiology in order to have an optimal separation between brain and systemic physiological effects. At the same time, the SPA-fNIRS approach also provides completely new insights into the interaction between brain activity and systemic physiology induced by a psychedelic. As psychedelics are affecting not only brain activity but also the physiological state of the whole body, an integrative physiological understanding of the physiological effects of psychedelics will require to investigate how the brain and the body are affected in parallel and how both interact—for example, changes in respiration will have an effect on the partial pressure of carbon dioxide in the arterial blood ($P_{a}{\mathrm{CO}}_{2}$), changing cerebral hemodynamics, as well as potentially interfering/modulating neurovascular-coupling.^144^^–^^146^ Therefore, possible $P_{a}{\mathrm{CO}}_{2}$ changes must be taken into account for a correct analysis and interpretation of fNIRS data^112^^,^^147^ (which, incidentally, also applies to fMRI data). Furthermore, changes in cerebral and extracerebral tissue hemodynamics induced by changes in the state of the autonomic nervous system need to be considered too.^148^^–^^150^ The SPA-fNIRS approach is an ideal method to explore these aspects.

What must also be taken into account is the possibility and already existing initial indications that psychedelics can alter neurovascular coupling, as has been shown, for example in rats for psilocin (the active metabolite of psilocybin).^151^ The authors rightly concluded that “caution is required when making inferences about drug effects on neuronal activity from changes detected in neuroimaging signals.” This is true for fMRI as well as fNIRS. More research is urgently needed to understand how psychedelics (in a dose- and substance-dependent manner) affect neurovascular coupling and vascular reactivity (e.g., ${\mathrm{CO}}_{2}$ cerebrovascular reactivity, cerebral autoregulation, and autonomic cerebrovascular reactivity) in humans. SPA-fNIRS is a useful technique in this case too.

Regarding the limited depth resolution of fNIRS, the inability to measure subcortical structures is of course a disadvantage, but it is clear from previous research that the cerebral cortex (which can be measured with fNIRS) is also always involved in psychedelic effects—for example, the prefrontal cortex (PFC) is particularly enriched in 5-HTA receptors expressed in the apical dendrites of layer 5 pyramidal neurons,^152^^–^^154^ the PFC $5\text{-}{\mathrm{HT}}_{2A}$ receptor occupancy correlates with the psychedelic effects of psilocybin in humans^45^^,^^155^ and prefrontal cortical areas activated by both psilocybin and ketamine.^28^^,^^30^^,^^36^^,^^37^^,^^47^ Moreover, networks of synchronized brain activity involve subcortical and cortical areas^156^^,^^157^ that change during the psychedelic state.^35^^,^^41^^,^^158^^,^^159^ Such changes in cortical network activity can, of course, also be analyzed with fNIRS neuroimaging.^87^^,^^160^^,^^161^ The principal disadvantage that only cortical areas can be measured with fNIRS is put into perspective by the fact that the cortex is also always influenced by psychedelics and brain activity also changes there.

## 7 Conclusion and Outlook

In summary, fNIRS is a neuroimaging method that has great potential for psychedelic research. It is expected that in the near future, the number of fNIRS studies investigating psychedelic effects in humans will increase rapidly, as the technique offers certain advantages over conventional hemodynamic-based neuroimaging techniques, enables novel study designs, and also has great potential to be used for multimodal neuroimaging (e.g., fNIRS in combination with fMRI, EEG, or PET).

fNIRS will be a good method to study cognitive control (with the PFC as an important brain region associated with and the multisource interference task as a typical test), attentional capacity, and possibly emotion processing, as well as the interaction between cognition and emotion, before, during and after psychedelic administration. fNIRS will also be well suited for longitudinal studies (which are currently scarce). In addition, fNIRS has great potential to investigate social interaction in a setting with psychedelics [e.g., fNIRS neuroimaging on the subject that got the psychedelic substance and in parallel on the person that monitors the subject and provides support when necessary (the “trip sitter”)].

At the same time, certain aspects must be taken into account when using fNIRS in order to carry out the measurements correctly, to optimally analyze the data and to correctly interpret the results physiologically. It is important to avoid misinterpretation of fNIRS data [e.g., confusion between extracerebral and cerebral components in the fNIRS signal or components caused by changes in systemic physiology (e.g., respiration or blood pressure)] with those induced by neurovascular coupling. Appropriate fNIRS hardware improvements and advanced signal processing methods are necessary to be applied and/or further developed. Good progress has already been made in this respect, and it is expected to accelerate enormously in the coming years, making the measurement and interpretation of fNIRS signals more reliable and accurate.

As far as the availability of commercially available fNIRS devices is concerned, the current situation is very good: there are many different commercially available fNIRS devices and NIRS oximeters, and more and more new companies and devices are entering the market. It is undoubted that fNIRS neuroimaging will be an integral part of the repertoire of modern neuroimaging.

Now is the time to exploit the momentum of the current resurgence of research on the effects of psychedelics and the momentum of the increasing progress and popularity of the fNIRS technique to establish fNIRS in psychedelic research. With this article, we hope to contribute to this development.
